# Norovirus-related chronic diarrhea in a patient treated with alemtuzumab for chronic lymphocytic leukemia

**DOI:** 10.1186/1471-2334-14-239

**Published:** 2014-05-06

**Authors:** Anne-Marie Ronchetti, Benoit Henry, Katia Ambert-Balay, Pierre Pothier, Justine Decroocq, Véronique Leblond, Damien Roos-Weil

**Affiliations:** 1 Hematology Department, Hôpital Pitié-Salpétrière, AP-HP, Université Pierre et Marie Curie Paris 06, GRC 11 (GRECHY), Paris, France; 2 Tropical and Infectious Diseases Department, Hôpital Pitié-Salpétrière, AP-HP, Université Pierre et Marie Curie, Paris, France; 3 Centre National de Référence des Virus Entériques, CHU de Dijon, Dijon, France

**Keywords:** Norovirus, Diarrhea, Immunosuppression, Alemtuzumab, Chronic lymphocytic leukemia

## Abstract

**Background:**

Norovirus infection is increasingly recognized as an important cause of persistent gastroenteritis in immunocompromised hosts and can be a potential cause of morbidity in these populations.

**Case presentation:**

Here, we report a case of norovirus-related chronic diarrhea occurring in a 62-year-old immunocompromised patient treated with alemtuzumab for chronic lymphocytic leukemia. Despite different therapeutic strategies including tapering of immunosuppressive therapy and immunoglobulin administration, diarrhea unfortunately did not resolve and lasted for a total of more than twelve weeks with prolonged norovirus fecal excretion.

**Conclusions:**

Norovirus infection can occur in the setting of alemtuzumab treatment, even as a single agent, and should be included in the differential diagnoses of acute and chronic diarrhea in these immunocompromised patients. Although the administration of oral immunoglobulin has been described as a promising efficient therapy, this was not the case in our patient. Clinical trials are thus clearly warranted to better define risk factors and efficient therapies for norovirus infection in immunocompromised populations.

## Background

Norovirus (NoV), a separate genus of the enteric virus family *Caliciviridae*, is actually recognized as the overall leading cause of acute gastro-enteritis (GE), being the first and second cause in adult and children, respectively, and accounts for more than 90% of GE outbreaks [1]. In immunocompetent individuals, symptoms usually last for a few days but recent studies have shown that NoV infection can be chronic and severe among vulnerable populations like solid organ and bone marrow transplantation (BMT) recipients [2-4], inherited immune deficiencies [5] or HIV infected patients [6]. Human mechanisms for protective immunity and clearance of NoV are not well defined. Although its precise mechanism is still unclear, it is hypothesized that cellular immunity plays an important role as suggested, for example, by the resolution of GE following the decrease of immunosuppressive therapy [3] or by the association between donor T cell recovery and NoV clearance [7] in BMT recipients.

Alemtuzumab is a humanized monoclonal IgG1 antibody directed toward the cell surface antigen CD52. It is largely used in chronic lymphoproliferative disorders like chronic lymphocytic leukemia (CLL), leading to profound T and B lymphocyte depletion and exposing patients to life-threatening opportunistic infections, particularly viruses, like CMV [8]. Although NoV infection has never been described in patients treated with alemtuzumab alone, the use of alemtuzumab in the conditioning regimen of BMT has been interestingly reported to be a potential risk factor for NoV GE in two studies [4,9].

NoV treatment in immunocompromised patients is challenging and mainly supportive as no specific therapy actually exists. Different strategies have been tested, from the adjustment of immunosuppressive treatment to the use of oral immunoglobulin (Ig) or antiviral drugs, but with controversial results [2]. We report here a case of NoV-related chronic diarrhea, in an immunocompromised patient treated with alemtuzumab, which did not respond to Ig administrations.

## Case presentation

**Figure 1:**
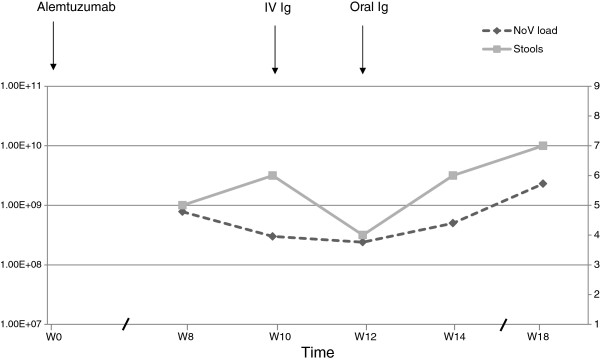
**Evolution of norovirus (NoV) loads and number of stools after alemtuzumab treatment.** NoV loads are represented in black dashed lines and expressed in log copies per g of stool. Stools are represented in gray solid lines and expressed in number per day. First, fecal samples have been weighed and then suspended in phosphate-buffered saline (pH 7.5) at a concentration of 10%. Viral RNA was extracted with a QIAamp viral RNA kit (Qiagen, Hilden, Germany). The NoVs were detected by RT-PCR on an ABI Prism 7500 Fast detector (Life Technologies) using the TaqMan One-Step PCR Master Mix reagent (Life Technologies) and previously published primers and probes for GI [18] and GII [10]. For quantitative assessment of fecal viral load, the number of NoV RNA copies was estimated by comparing the sample C_T_ value with standard curves. Among the five available samples, four were positive for GII.7 in the polymerase gene and for GII.6 in the capsid gene. Then, as described previously [10], the ORF1/ORF2 junction was sequenced using the same primers. The nucleotide sequences were aligned and compared to corresponding sequences of NoV strains available in the GenBank database using Fasta program. These sequences showed a homology of 92% and 98% for GII.7 and GII.6, respectively, and a 100% homology between them. Abbreviations: Ig, immunoglobulins; IV, intravenous; NoV, norovirus; W, week.

A French 62-year-old man was hospitalized in our institution for severe invasive aspergillosis. His medical history was remarkable for chronic lymphocytic leukemia (CLL), diagnosed in 1998 and first requiring therapy in 2006. From 2006 to 2010, our patient received several lines of treatment comprising polychemotherapies with anthracyclines (in 2006), purines analogs (fludarabine) (in 2007), alkylating agents (cyclophosphamide) (in 2010), high dose corticosteroids and immunotherapy (rituximab) (in 2006, 2007 and 2010), alternatively or in combination. Last relapse of CLL occurred in December 2011 and motivated a new therapeutic sequence with alemtuzumab and dexamethasone. In January 2012, three weeks after the initiation of alemtuzumab, he developed fever, cough and subacute vision loss of the left eye, revealing a multi-organ aspergillosis that involved lung, eyes and also brain. Apart from this severe infectious complication that required prolonged antifungal therapy with voriconazole, the clinical evolution during hospitalization was marked by the persistence of intermittent fever and the progressive onset of fluctuating watery diarrhea, which started six weeks after the first alemtuzumab dose (February 2012). Diarrhea lasted for a total of more than twelve weeks. Diarrheal stools were profuse but contained neither blood nor mucus. The patient had no concomitant abdominal pain, vomiting or myalgia. He had no recent travel history, no family history of vomiting or diarrhea and there was no argument for any nosocomial outbreak at this time in our department. All potential medications that can induce diarrhea, including voriconazole and alemtuzumab, were withdrawn but without efficacy. Empiric antibiotic therapies (successively ceftriaxone, ciprofloxacine, metronidazole and piperacillin/tazobactam) were also unsuccessful. Of note, repeated microbiological stool examinations, including cultures and assays for pathogenic bacteria (*C. difficile*, *Camplyobacter spp*., *Salmonella spp*., and *Yersinia spp.*) and standard detection of protozoans (including searches for *Microsporidium sp*., *Cryptosporidium sp*., *Isospora sp*. and *Giardia lamblia*), were negative. Serum cytomegalovirus (CMV) and adenovirus polymerase chain reactions (PCRs), stool rotavirus and adenovirus PCRs were also negative. Colonoscopy did not find any ulcerative lesion and pathologic examination of colonic biopsies any cytopathic effect. There was also no argument for aspergillosis involvement in the gastrointestinal tract. The persistence of diarrhea and the negativity of standard pathogenic microorganisms responsible for GE finally raised the possibility of NoV infection. Fecal NoV reverse transcriptase (RT)-PCR was positive for recombinant genogroup II, genotype 7 and genotype 6 (GII.7/II.6). Sequencing of the ORF1/ORF2 junction, as described previously [10], excluded the possibility of co-infection of two NoV genotypes. More, NoV viral loads have been retrospectively calculated for four fecal samples (see Figure 1). Values ranged from 2.4 × 10^8^ copies/g up to 2.3 × 10^9^ copies/g of stool. Our results are concordant with reported values in previous studies which ranged from 10^3^ to 10^11^ copies/g of stool, with median/mean values between 10^7^ and 10^8^ copies/g [11,12] and consistent with prolonged viral excretion.

At that time, the Ig level was low (IgG 5.6 g/L (normal: 6.7-12.5 g/L); IgA 0.25 g/L (normal: 1.04-3.33 g/L), IgM 0.57 g/L (normal 0.4-2.3 g/L)) and the lymphocyte count show profound T CD4 and CD8 depletion (CD4+ 0.03 G/L, CD8+ 0.05 G/L, B cell count of 0.9 G/L). Of note, two months before alemtuzumab treatment in October 2011, T CD4+/CD8+ counts were only slightly decreased at, respectively, 0.4 and 0.3 G/L. As the administration of parenteral or oral Ig has been suggested to be of potential interest in NoV GE after transplantation [11,13-15], we tried these two therapeutic options but without success (see Figure 1). First, intravenous Ig was administrated at the dose of 0.5 g/kg for a single day, ten weeks after the beginning of alemtuzumab and four weeks after the diarrhea onset. Two weeks later, facing up to the persistence of diarrhea, we instilled Ig by oral route through a nasogastric tube at the same total dose but fractioned in four doses, every six hours for one day. Profuse diarrhea persisted and five further fecal NoV RT-PCRs were still positive for NoV GII.7/II.6 in a time period of eight weeks. Finally, evolution was fatal due to uncontrolled *E. Coli* bacteriemia.

## Discussion

Noroviruses have been identified as an important cause of chronic diarrhea in immunocompromised hosts. Although there is growing number of case reports, it has never been described after the use of alemtuzumab as a single agent. Alemtuzumab is an anti-CD52 monoclonal antibody, which is used in CLL patients who failed fludarabine therapy and sometimes in frontline therapy in case of high-risk cytogenetic abnormalities. It has also been used in other circumstances, such as multiple sclerosis, or organ transplant rejection. The CD52 antigen is present on the surface of T, B and NK lymphocytes, and also on macrophages and dendritic cells. Alemtuzumab is a potent immunosuppressive therapy that can lead to a wide variety of severe infectious complications, especially viral and bacterial infections [15]. In patients receiving alemtuzumab, the lymphocytic depletion is estimated in median at 5 years for the CD4+ and 3 years for the CD8+ fraction [8]. In the case of our patient, the persistent NoV infection [15] despite the tapering of alemtuzumab also illustrates the very long lasting lymphocyte depletion due to this molecule, making a rapid diagnosis of infectious complications due to alemtuzumab more suitable. Although its precise role could not be certain in our observation, some arguments plead for the potential involvement of alemtuzumab in the onset of NoV infection. First, NoV diarrhea began six weeks after initiation of alemtuzumab, while the last immunosuppressive therapies (rituximab plus cyclophosphamide) have been administrated more than a year ago. Second, alemtuzumab was in a large extent responsible for the profound T-cell depletion as T-cell counts were near normal before its start. Moreover, as described above, the use of alemtuzumab is associated with severe infectious complications and has been recognized as a potential risk factor for NoV GE in allografted children when used in the conditioning regimen [9]. NoV-related chronic diarrhea has also already been reported in the setting of hypogammaglobulinemia and after immunotherapy as it has been described in a CLL patient treated with rituximab [16].

Despite different therapeutic strategies, diarrhea did not resolve in the case of our patient and NoV viral loads in fecal samples remained positive. The most promising approach reported in the literature is the use of enteral Ig as it has been described successful in four immunocompromised patients: two children with small bowel transplantation [13] and two adults, one with cardiac [17] and the other with renal transplantation [11]. The failure of this strategy in our patient could be due to the profound level of immunosuppression and/or the mode of Ig administration (rhythm, period) although we administrated the same total dose as in reported successful experiences.

## Conclusions

NoV treatment in immunocompromised patients is challenging as no specific antiviral agent actually exists and as the tapering of immunosuppressive drugs is not always possible. Vaccine research is ongoing, but no vaccine is currently available. Although parenteral and oral Ig administrations have been reported to be efficient, it was not the case in our patient. Profound T cell depletion and hypogammaglobulinemia may explain this failure of NoV clearance. Given the prolonged survival of patients with hematological malignancies and the increasing use of immunotherapies, it is likely that there will be more reports of NoV infections. NoV should be included in the differential diagnoses of acute and chronic diarrhea in immunocompromised patients and clinical trials should also be developed to define risk factors and efficient therapies.

## Consent

Written informed consent was obtained from the family of the patient for publication of this Case report. A copy of the written consent is available for review by the Editor of this journal.

## Competing interests

The authors declare that they have no competing interests.

## Authors’ contributions

AMR and DRW took care of the patient, collected and analyzed data and wrote the manuscript. BH, JD and VL took care of the patient and critically revised the manuscript. KAB and PP performed the molecular genetic studies and critically revised the manuscript. All authors read and approved the final manuscript.

## Pre-publication history

The pre-publication history for this paper can be accessed here:

http://www.biomedcentral.com/1471-2334/14/239/prepub
